# The Association of 5-HT2A, 5-HTT, and LEPR Polymorphisms with Obstructive Sleep Apnea Syndrome: A Systematic Review and Meta-Analysis

**DOI:** 10.1371/journal.pone.0095856

**Published:** 2014-04-22

**Authors:** Baodong Qin, Zhen Sun, Yan Liang, Zaixing Yang, Renqian Zhong

**Affiliations:** Department of Laboratory Diagnostics, Changzheng Hospital, Second Military Medical University, Shanghai, China; Charité – Universitätsmedizin Berlin, GERMANY

## Abstract

**Objective:**

A consensus has not been reached regarding the association of several different gene polymorphisms and susceptibility to obstructive sleep apnea syndrome (OSAS). We performed a meta-analysis to better evaluate the associations between 5-HT2A, 5-HTT, and LEPR polymorphisms, and OSAS.

**Method:**

5-HT2A, 5-HTT, and LEPR polymorphisms and OSAS were identified in PubMed and EMBASE. The pooled odd rates (ORs) with 95%CIs were estimated using a fixed-effect or random-effect models. The associations between these polymorphisms and OSAS risk were assessed using dominant, recessive and additive models.

**Results:**

Twelve publications were included in this study. The -1438 “A” allele of 5-HT2A was identified as a candidate genetic risk factor for OSAS (OR: 2.33, 95%CI 1.49–3.66). Individuals carrying the -1438 “G” allele had a nearly 70% reduced risk of OSAS when compared with AA homozygotes (OR: 0.30, 95%CI 0.23–0.40). There was no significant association between 5-HT2A 102C/T and OSAS risk, using any model. The “S” allele of 5-HTTLPR conferred protection against OSAS (OR: 0.80, 95%CI 0.67–0.95), while the “10” allele of 5-HTTVNTR contributed to the risk of OSAS (OR: 2.08, 95%CI: 1.58–2.73). The “GG” genotype of LEPR was associated with a reduced risk of OSAS (OR: 0.39, 95%CI 0.17–0.88).

**Conclusion:**

The meta-analysis demonstrated that 5-HTR-1438 “A” and 5-HTTVNTR “10” alleles were significantly associated with OSAS. The “S” allele of 5-HTTLPR and the “GG” genotype of LEPR conferred protection against OSAS. Further studies, such as Genome-Wide Association study (GWAS), should be conducted in a large cohort of OSAS patients to confirm our findings.

## Introduction

Obstructive sleep apnea syndrome (OSAS) is a respiratory disorder characterized by upper airway obstruction during sleep, breathing pauses with oxygen desaturation, and arousal from sleep. OSAS is the third most prevalent respiratory disease after asthma and chronic obstructive pulmonary disease [1]. It has been estimated that about 2–4% of middle-aged people are affected by OSAS. OSAS is recognized as a chronic, complex disease related to metabolic syndrome, cardiovascular disease, neurocognitive and mood disorders [2]. With the rise in overweight and obesity, the prevalence of OSAS should increase, representing a serious public-health problem with substantial social and economic costs [3].

Although the pathogenetic mechanism of OSAS remains unclear, many factors have been incriminated. A combination of genetic makeup and environmental factors could contribute to the development of OSAS. The high concordance of first-degree relatives with OSAS, family clustering, and ethnic differences show that genetic factors may play an important role in the pathogenesis of OSAS [4]. It has been reported that genetic factors could account for about 40% of the variance in the apnea-hypopnea index (AHI) of OSAS patients [5]. It is currently thought that multiple gene interactions in a suitable environment may lead to OSAS [6].

Evaluation of the genetic contribution to the occurrence of OSAS is of interest [7]. Several polymorphisms have been suggested to play a role and a number of family and SNP studies have been performed. Serotonin (5-hydroxytryptamine; 5-HT) regulates a variety of physiological functions through 5-HT receptors (5-HT2A, 5-HT2B, 5-HT2C). These play a critical role in the patency of the upper airway and the prevention of glossocoma. A functional gene polymorphism of 5-HT2A has been identified to alter gene transcription, thus influencing the expression level of the receptor [8]. Polymorphisms in the 5-HT2A gene, characterized by reduction of receptor number and serotonin concentration in postsynaptic neurons, were associated with the development of OSAS [9], [10]. 5-HT reuptake is mediated by the serotonin transporter (5-HTT) [11], [12]. Polymorphisms of this gene could lead to alterations in 5-HT concentrations. Two such polymorphisms (variable number tandem repeat (VNTR) and 5-HTTLPR) have been described [13], [14]. Uptake of serotonin in cells with the “L/L” 5-HTTLPR genotype was more than that in cells carrying the “S/L” or “S/S” genotypes. The S allele was responsible for low uptake activity. Leptin is an adipocyte-derived hormone which plays an important role in metabolic control. The potential associations of leptin and LEPR gene polymorphisms with OSAS have been assessed in different populations [15]. Although the relationship between 5-HT2A, 5-HTT, LEPR and OSAS has been evaluated, the results were not consistent. To date, no large-scale Genome-Wide Association study (GWAS) has been performed. We performed a meta-analysis to better evaluate the association between reported polymorphisms and OSAS risk.

## Methods

### Search Strategy

We searched PubMed and EMBASE in all languages for relevant studies. The final date for inclusion was March 10, 2013. Comprehensive search themes included Medical Subject Headings (MeSH) terms and keywords: “sleep apnea”; “obstructive sleep apnea Syndrome”; “sleep apnea/hypopnea syndrome” and “serotonin 2A receptor(5-HT2A)”; “serotonin transporter protein(5-HTT)”; “leptin receptor (LEPR)”; “polymorphism”; ”variant”; and “genotype”.

### Study selection

Inclusion criteria for this study were: (1) OSAS patients must be clearly diagnosed; (2) the study must be a case-control study; (3) the study should report enough genotyping information to extract and estimate odds ratios (ORs) with a 95% confidence interval (CI). Studies were excluded if: (1) the diagnosis was unclear; (2) the study did not contain sufficient data for extraction; (3) the study design was based on case, family or sibling pairs; or (4) no control group was included in the study. When overlapping reports occurred, the study with largest number of patients was selected.

### Data extraction

All included studies were retrieved and the required information was extracted separately, in duplicate, by two authors (BDQ and SZ). Any discrepancies were resolved by discussion and agreement. The characteristics collected from each study were: author, year of publication, country, age, AHI, sample size, diagnosis criteria, genotype, allele frequency, and specific technique of analysis. If the allele frequency was not given, it was calculated from the corresponding genotype distributions. The OR with its 95%CI was extracted or calculated for each study.

### Assessment of study quality

There is no standard quality criteria for single nucleotide polymorphism (SNP) studies, so we employed a modified Newcastle-Ottawa scale (NOS) score system to assess the quality of these non-randomized studies [16]. A total of eight items are included in the NOS. These are divided into three categories describing selection, comparability and exposure. A maximum of two stars was given for each of these items. A study awarded 0–3 stars was classified as a low quality study, while 4–6 stars and 7–9 stars were moderate and high quality studies, respectively [17].

### Statistical analysis

We performed a systematic review and meta-analysis following a predetermined protocol in accordance with the Meta-analysis of Observational Studies in Epidemiology (MOOSE) reporting guidelines [18]. A meta-analysis was performed of polymorphisms of the 5-HT2A, 5-HTT, and LEPR genes, to examine their association with OSAS using additive, recessive and dominant models. The pooled ORs with 95%CIs were calculated in random-effects or fixed-effects models to measure the strength of the associations between these polymorphisms and OSAS risk. Heterogeneity of effects across studies was evaluated using the means of χ^2^-based Q test and I^2^ test. P<0.10 was considered to be representative of significant heterogeneity with the Q test. The I^2^ statistic represented quantification of heterogeneity ranging from 0% to 100%. Hundred percent represented a high degree of heterogeneity and 50%-<100% represented substantial heterogeneity [19]. If there was significant heterogeneity (P<0.10 for Q test; I^2^>50% for I^2^ test), a random-effects model was used to pool the data. Otherwise, a fixed-effects model was used. A funnel plot was employed to visually assess potential publication bias in meta-analyses including more than five studies. The Egger's regression asymmetry test and Begg's rank correlation test were used to statistically examine publication bias. A sensitivity analysis was performed to assess the impact of each individual study upon the overall ORs, using the one-study removal approach [20]. Hardy-Weinberg Equilibrium was assessed using χ2 tests for the polymorphism investigated in each study. All analyses were conducted using STATA 11.0 (StataCorp, College Station TX, USA). P<0.05 was considered statistically significant.

## Result

### Studies selection

**Figure 1:**
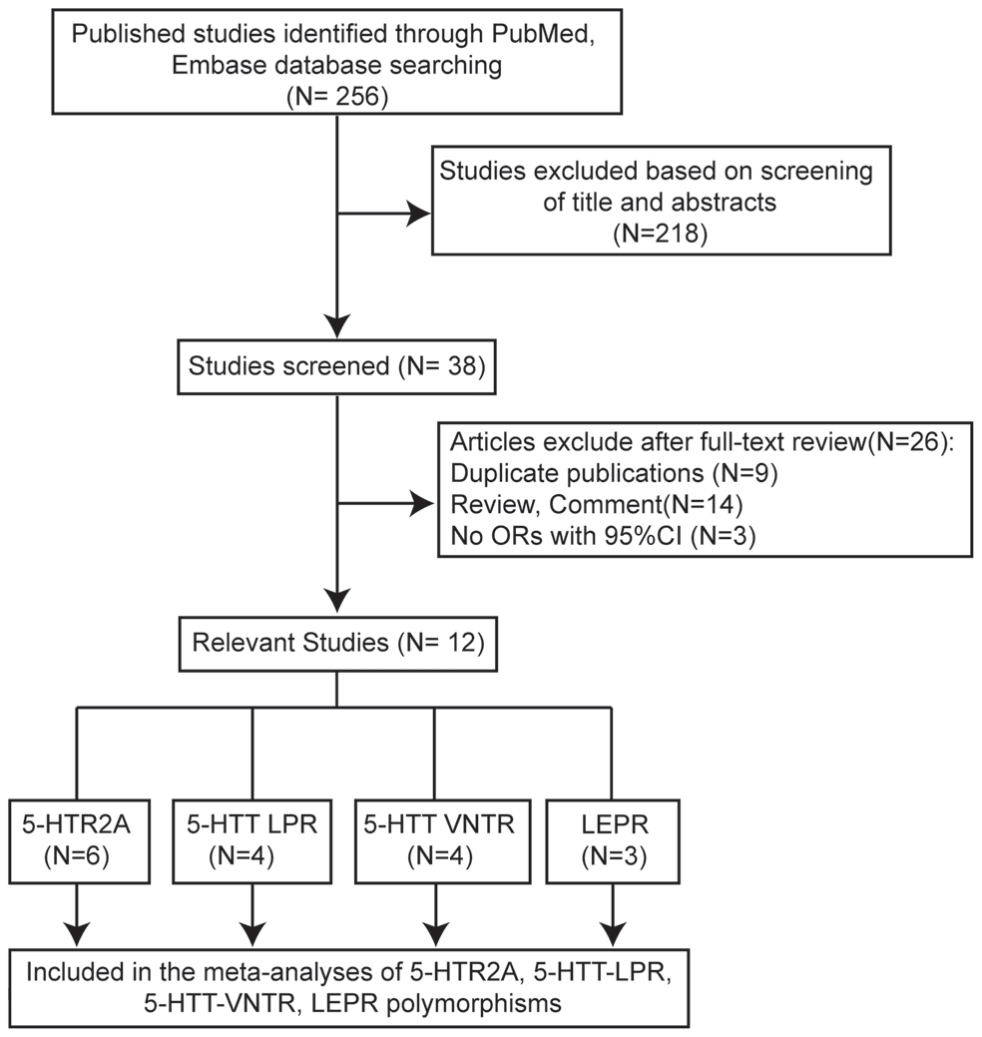
The flowchart showing articles identification, inclusion and exclusion.

Two hundred fifty-six non-overlapping articles were identified in the initial published work search. Two hundred eighteen articles were excluded based on screening of abstracts or titles. Thirty-eight full-text articles were retrieved and assessed for eligibility. Twenty-six of these articles were excluded (**Figure 1**), leaving twelve eligible studies in this present study.

### Characteristics of included studies

Nine of the twelve studies included were in English and three in were Chinese. One thousand twenty OSAS patients and one thousand eighty-three control subjects were reported [9], [10], [13]–[15], [21]–[27]. Six of the twelve studies were conducted to clarify the association between 5-HT2A polymorphisms and OSAS. These studies included 728 OSAS patients and 566 controls subjects. Four studies evaluated 5-HTT polymorphisms and included 521 OSAS patients and 755 control subjects. Three studies evaluated the LEPR polymorphism and included 292 OSAS patients and 167 control subjects. ORs with 95% CIs were extracted from each study. A database was established for the information or characteristics extracted from these studies (**Table 1** and **Table 2**). The 12 reports included patients of American (n = 1), Asian (n = 8), and European (n = 3) ethnicity. The number of patients in each report ranged from 100 to 592. The diagnosis of OSAS was based on symptoms and polysomnography (PSG). Hardy-Weinberg equilibrium testing showed deviation from HWE in some of the studies (**Table S1**).

**Table 1:** Characteristic of the included studies about the association between 5-HT2A polymorphism and OSAS risk.

	Author	Year	Country	Age(Case/Control)	AHI(Case/Control)	Case/Control	Diagnosis	Genotype Method	Genotype Frequency		
									Genotype	OSAS	Control
102C/T	Yin, et al	2011	China	41.4±9.2**_⊢_**	42.3±22.1**_⊢_**	210/105	PSG AHI ≥5 events/h	PCR	TT	52	30
				40.6±10.4**_⊤_**	2.3±1.6**_⊤_**				CT	107	51
									CC	51	24
	Sakai, et al	2005	Japan	48.3±10.1	46.1±25.8	177/100	PSG AHI ≥5 events/h	PCR	TT	47	25
				38. ±11.1	NS				CT	90	46
									CC	40	29
	Bayazit, et al	2006	Turkey	NS	NS	55/102	PSG	PCR	TT	17	28
				NS	NS		Muller Maneuver		CT	21	54
							Laryngoscopy		CC	17	20
	De Carvalho, et al	2013	brazil	50.6±11.1	32.6±25.7	100/100	PSG AHI ≥5 events/h	PCR	TT	23	20
				44.6±12.0	1.9±1.5				CT	66	69
									CC	11	11
	Zhu, et al	2007	China	44.2±2.0	NS	65/54	PSG	PCR	TT	18	15
				43.0±2.1	NS				CT	30	28
									CC	17	11
	Chen, et al	2013	China	43.8±3.0	42.6±14.8	121/105	PSG AHI ≥5 events/h	PCR	TT	34	29
				43.0±2.7	3.7±1.3		AHI ≥15 events/h		CT	56	54
									CC	31	22
-1438 G/A	Yin, et al	2011	China	41.4±9.2	42.3±22.1	210/105	PSG AHI ≥5 events/h	PCR	AA	85	17
				40.6±10.4	2.3±1.6				AG	82	67
									GG	43	21
	Bayazit, et al	2006	Turkey	NS	NS	55/102	PSG	PCR	AA	24	25
				NS	NS		Muller Maneuver		AG	29	50
							Laryngoscopy		GG	12	27
	Piatto, et al	2011	brazil	50.6±11.1	32.6±25.7	100/100	PSG AHI ≥5 events/h	PCR	AA	35	19
				44.6±12.0	1.9±1.5				AG	61	71
									GG	4	10
	Zhu, et al	2007	China	44.2±2.0	NS	65/54	PSG	PCR	AA	41	15
				43.0±2.1	NS				AG	15	14
									GG	9	25
	Chen, et al	2013	China	43.8±3.0	42.6±14.8	121/105	PSG AHI ≥5 events/h	PCR	AA	74	28
				43.0±2.7	3.72±1.28				AG	30	28
									GG	17	49

NS: None Stated.

⊢: Indicated age of OSAS patients group in these studies.

**⊤**: Indicated age of Control group in these studies.

**Table 2:** Characteristic of the included studies about the associations between 5-HTT, LEPR polymorphisms and OSAS risk.

	Author	Year	Country	Age(Case/Control)	AHI(Case/Control)		Case/Control	Diagnosis	Genotype Method	Genotype Frequency		
										Genotype	OSAS	Control
5-HTT	Chen, et al	2013	China	43.8±3.0_⊢_		42.6±14.8_⊢_	121/105	PSG AHI ≥5 events/h	PCR)	SS	59	55
LPR				43.0±2.7**_⊤_**		3.7±1.3**_⊤_**		AHI ≥15 events/h		SL	35	34
										LL	27	16
	Ylmaz, et al	2005	Turkey	NA		NS	42/162	PSG	PCR	SS	6	60
				NA		NS				SL	16	71
										LL	4	35
	Yue, et al	2008	China	45.2±11.8		53.9±16.4	254/338	PSG AHI ≥5 events/h	PCR	SS	114	173
				43.2±12.7		NS				SL	106	131
										LL	34	34
	Yue, et al	2005	China	42.3±10.1		NS	104/150	PSG AHI ≥10 events/h	PCR	SS	51	78
				NA		NS				SL	33	54
										LL	20	18
5-HTT	Chen, et al	2013	China	43.8±3.0		42.6±14.8	121/105	PSG AHI ≥5 events/h	PCR	10/10	8	1
VNTR				43.0±2.7		3.7±1.3		AHI ≥15 events/h		10/12	24	10
										12/12	89	94
	Ylmaz, et al	2005	Turkey	NA		NS	42/162	PSG	PCR	10/10	3	14
				NA		NS				10/12	13	61
										12/12	9	93
	Yue, et al	2008	China	45.2±11.8		53.9±16.4	254/338	PSG AHI ≥5 events/h	PCR	10/10	6	3
				43.2±12.7		NS				10/12	46	41
										12/12	202	294
	Yue, et al	2005	China	42.3±10.1		NS	104/150	PSG AHI ≥10 events/h	PCR	10/10	3	1
				NA		NS				10/12	20	14
										12/12	81	135
LEPR	Popko, et al	2007	Poland	21–77		>5	102/77	PSG AHI ≥5 events/h	PCR assay	Gln/Gln	18	26
				18–65		<5				Gln/Arg	61	40
										Arg/Arg	23	11
	Hanaoka,	2008	Japan	50.3±2.0		49.1±3.9	130/50	PSG AHI ≥10 events/h	PCR	Gln/Gln	63	21
	et al			50.0±4.4		2.1±0.5				Gln/Arg	57	28
										Arg/Arg	5	1
	Huang,	2003	China	43.4±0.8		NS	60/40	PSG	PCR-RFLP	Gln/Gln	79	64
	et al			43,9±1.2		NS				Gln/Arg	22	23
										Arg/Arg	2	1

NS: None Stated.

⊢: Indicated age of OSAS patients group in these studies.

**⊤**: Indicated age of Control group in these studies.

### 5-HT2A 102 C/T and OSAS

Six studies evaluated the association between 5-HT2A 102C/T and OSAS. The pooled data revealed no significant association between 5-HT2A 102C/T polymorphism and OSAS risk, using any model (**Table 3**). The frequency of the minor “C” allele was not significantly different in OSAS patients and controls (OR: 0.97, 95%CI 0.83–1.14), suggesting that the polymorphism was not linked to OSAS development. There was no significant heterogeneity in this meta-analysis. Subgroup meta-analysis stratified by ethnicity found no significant association with Caucasian or Asian populations.

**Table 3:** Meta-analysis of associations between 5-HT2A, 5-HTT, LEPR polymorphisms and OSAS in the additive, dominant and recessive Models.

SNP	Comparison	OR with 95%CI		Heterogeneity			Publication Bias	
			χ2	Q test	I^2^	Tau2	Begg's test	Egger's test
5-HT2A 102 C/T	T vs C	0.97(0.83–1.14)	2.02	0.846	0.00%	0	0.707	0.449
	TT vs CT	1.07(0.81–1.40)	1.98	0.852	0.00%	0	0.133	0.087
	TT vs CC	0.93(0.67–1.29)	2.07	0.839	0.00%	0	1.000	0.699
	CC vs CT	1.13(0.84–1.50)	5.63	0.344	11.20%	0.017	0.452	0.213
	TT+CT vs CC	0.90(0.69–1.19)	4.67	0.458	0.00%	0	1.000	0.316
	CC+CT vs TT	0.98(0.76–1.26)	1.06	0.957	0.00%	0	0.707	0.263
5-HT2A-1438 G/A	A vs G	2.33(1.49–3.66)	22.9	0	82.60%	0.217	0.462	0.458
	AA vs GG	4.22(2.38–7.49)	8.49	0.075	52.90%	0.222	1.000	0.989
	GG vs AG	0.66(0.31–1.40)	15.2	0.004	73.60%	0.524	0.806	0.296
	AA vs AG	2.78(2.03–3.81)	2.33	0.675	0.00%	0	0.806	0.543
	AA+AG vs GG	2.44(1.11–5.38)	20.8	0	80.70%	0.637	0.806	0.631
	GG+AG vs AA	0.30(0.23–0.40)	3.53	0.473	0.00%	0	0.806	0.747
5-HTTLPR	S vs L	0.80(0.67–0.95)	0.07	0.995	0.00%	0	1.000	0.435
	SS vs LL	0.65(0.46–0.93)	0.27	0.966	0.00%	0	1.000	0.396
	SS vs SL	0.86(0.67–1.11)	2.76	0.430	0.00%	0	0.713	0.713
	SL vs LL	0.77 (0.54–1.11)	3.60	0.308	16.70%	0.031	0.734	0.547
	SS+SL vs LL	0.72(0.51–1.01)	0.22	0.977	0.00%	0	0.734	0.285
	LL+SL vs SS	1.25(0.99–1.59)	0.98	0.805	0.00%	0	0.734	0.557
5-HTTVNTR	10 vs 12	2.08(1.58–2.73)	3.31	0.346	9.40%		0.089	0.278
	10/10 vs 12/12	3.75(1.67–8.45)	1.29	0.731	0.00%	0	0.734	0.135
	10/10 vs 10/12	1.67(0.74–3.77)	0.94	0.816	0.00%	0	0.308	0.236
	10/12 vs 12/12	1.97(1.42–2.73)	1.35	0.718	0.00%	0	0.734	0.090
	10/10+10/12 vs 12/12	2.14(1.57–2.91)	2.10	0.551	0.00%	0	0.734	0.187
	10/10 vs 12/12+10/12	3.00(1.39–6.46)	1.89	0.596	0.00%	0	0.308	0.141
LEPR	G vs A	0.78(0.58–1.05)	3.73	0.155	46.40%	0.009	1.000	0.791
	GG vs AA	0.39(0.17–0.88)	0.40	0.818	0.00%	0	1.000	0.042
	GG vs AG	0.80(0.40–1.59)	5.62	0.060	64.40%	0.241	1.000	0.517
	AA vs AG	1.46(0.71–3.04)	0.27	0.875	0.00%	0	1.000	0.703
	AA+AG vs GG	1.32(0.65–2.67)	6.11	0.047	67.30%	0.260	1.000	0.652
	GG+AG vs AA	0.57(0.28–1.15)	0.03	0.984	0.00%	0	1.000	0.958

### 5-HT2A-1438 G/A and OSAS

**Figure 2:**
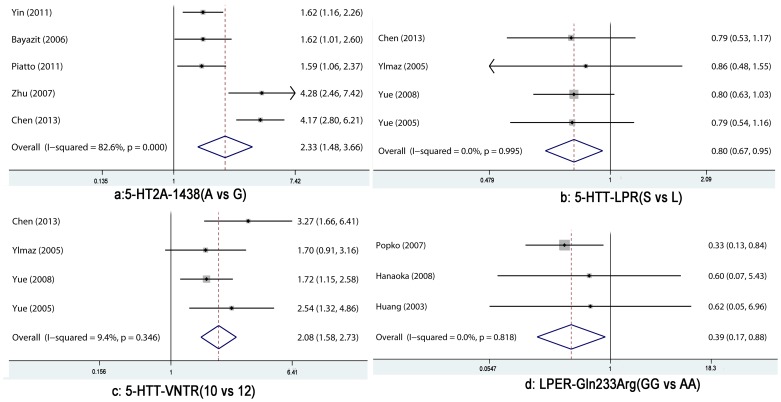
Forest plot of the association between 5-HT2A-1438(A *vs*. G), 5-HTT-LPR(S *vs*. L), 5-HTT-VNTR (10 *vs*. 12), LEPR-Gln223Arg (GG *vs*. AA) and OSAS risk in the overall population (a: Meta-analysis with a random-effects model; b, c, d: Meta-analysis with a fixed-effects model).

Five studies evaluated the association between -1438 G/A polymorphism and OSAS. Dominant and recessive models were examined. Individuals carrying the “G” allele (AG+GG) had a nearly 70% reduced risk for the development of OSAS, when compared with AA homozygotes (OR: 0.30, 95%CI 0.23–0.40). Individuals with the “A” allele were more susceptible to OSAS than GG homozygotes (OR: 2.44, 95%CI 1.11–5.38). Using the additive model, a significant association was found (OR: 2.78, 95%CI 2.03–3.81 for AA *vs*. AG; OR: 4.22, 95%CI 2.38–7.50 for AA *vs*. GG). AA carriers had an increased risk of OSAS compared with individuals having an AG or GG genotype. Significant heterogeneity was observed in the recessive and additive models (**Table 3**). The -1438 “A” allele was a candidate genetic risk factor for OSAS (OR: 2.33, 95%CI 1.49–3.66). There was significant heterogeneity across the studies with respect to the association between the “A” allele and OSAS risk (p<0.05, I^2^ = 82.60%) (**Figure 2**). The association was much stronger in Asian patients than in Caucasian patients. Assessment of bias indicated that heterogeneity was mainly found across the studies based on Asian populations rather than Caucasian populations. This supports the finding that ethnicity may contribute to the high degree of heterogeneity. Due to the small number of included studies, subgroup meta-analyses and assessment of bias by other factors could not be conducted.

### 5-HTT-LPR and OSAS

Four studies evaluated the association of 5-HTT-LPR polymorphisms with OSAS (**Table 2**). The meta-analysis revealed that the “S” allele conferred protection against OSAS (OR: 0.80, 95%CI 0.67–0.95). Individuals with the “S” allele had a 20% lower risk of OSAS than individuals with the “L” allele (**Figure 2**). The additive model demonstrated a greater association of the “SS” genotype with OSAS than that of the “LL” genotype (OR: 0.65, 95%CI 0.46–0.93). There was no significant heterogeneity across the studies. The subgroup meta-analysis of ethnicity could not be performed due to the small sample size.

### 5-HTT-VNTR and OSAS

Four studies evaluated the relationship of 5-HTT-VNTR polymorphisms with OSAS. The “10” allele was significantly associated with OSAS (OR: 2.08, 95%CI: 1.58–2.73) (**Figure 2**). The additive, recessive, and dominant models all showed a significant increased risk of OSAS among individuals with the “10” allele. The positive association was strongest when individuals with the “10/10” genotype” were compared with those carrying the “10/12” genotype (OR: 3.75, 95%CI: 1.7–8.45). No significant heterogeneity was found across these 4 studies.

### LEPR- Gln223Arg and OSAS

Three studies evaluated the relationship between LEPR- Gln223Arg and OSAS. The “GG” genotype” was associated with a low risk for OSAS, when compared with the “AA” homozygote (OR: 0.39, 95%CI 0.17–0.88) (**Figure 2**). A significant association was not found using recessive and dominant models. The risk of having OSAS in individuals with the “G” allele was 0.78. This was not significantly different from the “AA” allele (95%CI 0.58–1.05).

### Sensitivity analysis

Sensitivity analyses were performed when more than 3 studies were involved. The stability and reliability of pooled ORs were examined using the leave-one-out method. This method repeated the meta-analysis after sequential exclusion of each study. The estimated ORs were not significantly influenced by any individual study.

### Publication bias

The funnel plots for associations between 5-HT2A 102C/T, 5-HT2A-1438G/A, and OSAS did not visually demonstrate significant publication bias (**Data not shown**). Both Begg's test and Egger's tests demonstrated no publication bias (**Table 3**).

### Study quality

The NOS scoring system was used to evaluate study quality. Five studies scored 8 stars, 5 studies scored 7 stars, 1 study scored 6 stars, and 1 study scored 5 stars (**Table S2**).

## Discussion

The contribution of genetic factors to the development of OSAS is supported by the increased risk of OSAS in first-degree relatives and siblings of OSAS patients. The association between genetic polymorphisms and susceptibility to OSAS remains poorly defined due conflicting data. We conducted a systematic review of published studies investigating the role of 5-HT2A, 5-HTT, and LEPR polymorphisms in OSAS and performed a meta-analysis of these studies. 5-HT2A-1438G/A, 5-HTT-LPR, 5-HTT-VNTR, and LEPR-Gln223Arg were associated with the development of OSAS. 5-HT2A-1438 “A” and 5-HTT-VNTR “10” alleles were genetic risk factors for OSAS, while the 5-HTT-LPR “S” allele was a low risk factor for OSAS. Individuals with the “GG” LEPR-Gln223Arg genotype had a lower risk of OSAS compared to those with the “AA” genotype.

Twelve studies published between 2003 and 2013 were included in this study. Ten studies were of high quality, 2 of medium quality and no study was of low quality. These findings support the results of this study as a meaningful analysis of the available data.

The association of SNPs in the 5-HT2A, 5-HTT, and LEPR genes with OSAS has been reported with conflicting results. There is no previous meta-analysis of these reports. We found no association between 5-HT2A and 102T/C, and OSAS. The -1438G/A polymorphism of the 5-HT2A gene was associated with the development of OSAS. The -1438 “A” allele was associated with OSAS. This positive association was observed in all reports we evaluated. The “AA” genotype of 5-HT2A-1438G/A was over-represented in OSAS patients. Individuals carrying the “AA” genotype had a greater risk of OSAS than those with the “GG” genotype (OR  = 4.22). Bayazit et al. did not identify this positive association between “AA” genotype and OSAS risk. Yin found that the “AA' genotype was over-expressed in patients with LSaO_2_ ≤75%. Both Chen et al. and Bayazit et al. found the frequency of “AA” genotype to be significantly higher in male OSAS patients. There were not enough patients in the studies we reviewed to perform these subgroup analyses.

The LPR VNTR polymorphism in the 5-HTT gene correlated with the occurrence of OSAS in some, but not all, studies. A meta-analysis was performed to better evaluate the association between LPR VNTR polymorphisms and OSAS. The “S” allele of 5-HTT-LPR and “SS” genotype was associated with a lower frequency of OSAS. The frequency of the “10” allele in the 5-HTT-VNTR was significantly higher in OSAS patients, suggesting that it was a risk factor for OSAS. These findings were consistent with those of Chen et al. and Yue et al., but not Yilmaz et al. Another observation made here was that the frequency of the “GG” genotype in exon 6 of the LEPR gene was significantly lower in OSAS patients than controls, suggesting this genotype was low risk factor.

Polymorphisms related to the development of OSAS mainly lie in the serotoninergic system, an important component of sleep and airway function during sleep. *In vitro* studies have shown that polymorphisms of the 5-HTR 2A/2C genes influence receptor expression [28]. These findings give better insight into the development of OSAS and physiopathology of the disorder [29].

The GWAS has given better insight into the genetic nature of human disease, and identified several disease specific genes. The small sample size of previous studies and of this meta-analysis show the need for GWASs of large numbers of OSAS patients in order to better detect genetic associations. The GWAS approach could detect new disease genes, give clues to the pathogenesis of OSAS, and provide indicators for diagnosis, treatment, and prevention of the disease.

There were several limitations to this study. The number of included studies was relatively small, restricting the power of the study. Previous studies have shown associations between gender, SaO2, severity of disease and gene polymorphisms. The small number of patients identified in our literature review did not allow subgroup meta-analysis. The publications included in this study designed to evaluate the roles of different gene polymorphisms in OSAS. These different study designs could be a potential source of bias. Ongoing, unpublished and missing studies from the literature review could have contributed to publication bias, although the funnel plots, Egger' test and Begg's test did not identify any bias. Finally, a combination of susceptibility genes and environmental factors could contribute to the development of OSAS. Our study could not evaluate gene-gene and gene–environment interactions due to the small number of patients identified. These findings need to be studied in a large cohort of OSAS patients in the future.

In conclusion, our study demonstrated that 5-HT2R, 5-HTT, and LEPR polymorphisms were significantly associated with the development of OSAS. 5-HT2A-1438 “A” and 5-HTT-VNTR “10” were independent risk factors for OSAS. The “S” allele of 5-HTT-LPR and the “GG” genotype of Gln223Arg in the LEPR gene conferred protection against OSAS. The small sample size may have affected the accuracy of our findings. GWAS is needed to better detect candidate disease genes and to expand our understanding of the genetic background of OSAS. Future research should also focus on the clinical relevance of these findings.

## Supporting Information

Table S1 The HWE test for ACE(I/D), TNF-α-308 A/G, 5-HT2A-102C/T, 5-HT2A-1438G/A, 5-HTT LPR, 5-HTT VNTR, LEPR genotype distribution in included studies.
(DOC)

Table S2 Methodological quality of included studies according to the NEWCASTLE-OTTAWA Quality Assessment Scale.
(DOCX)

Checklist S1 PRISMA Checklist.
(DOC)
