# Supplementary material for: The Association of 5-HT2A, 5-HTT, and LEPR Polymorphisms with Obstructive Sleep Apnea Syndrome: A Systematic Review and Meta-Analysis
Source: PLoS One. 2014 Apr 22;9(4):e95856. doi: 10.1371/journal.pone.0095856 (PMC3995918; doi:10.1371/journal.pone.0095856)
Supplement: Table S1 — The HWE test for ACE(I/D), TNF-α-308 A/G, 5-HT2A-102C/T, 5-HT2A-1438G/A, 5-HTT LPR, 5-HTT VNTR, LEPR genotype distribution in included studies. (DOC) [file pone.0095856.s001.doc]

Supplementary. Table 1: The HWE test for ACE(I/D), TNF-α-308 A/G, 5-HT2A-102C/T, 5-HT2A-1438G/A, 5-HTT LPR, 5-HTT VNTR, LEPR genotype distribution in included studies.

| Polymorphism | Author | Year | OSAS Group | Control Group |
| --- | --- | --- | --- | --- |
| 5-HT2A-102C/T | Yin | 2011 | 0.782 | 0.795 |
|  | Sakai | 2005 | 0.805 | 0.432 |
|  | Bayazit | 2006 | 0.08 | 0.509 |
|  | Piatto | 2011 | <0.05 | <0.05 |
|  | Zhu | 2007 | 0.536 | 0.753 |
|  | Chen | 2013 | 0.417 | 0.734 |
| 5-HT2A-1438G/A | Yin | 2011 | 0.007 | 0.004 |
|  | Bayazit | 2006 | 0.539 | 0.846 |
|  | Piatto | 2011 | <0.05 | <0.05 |
|  | Zhu | 2007 | <0.05 | <0.05 |
|  | Chen | 2013 | <0.05 | <0.05 |
| 5-HTT LPR | Chen | 2013 | <0.05 | <0.05 |
|  | Yılmaz | 2005 | 0.225 | 0.108 |
|  | Yue | 2008 | 0.242 | 0.218 |
|  | Yue | 2005 | <0.05 | 0.08 |
| 5-HTT VNTR | Chen | 2013 | <0.05 | 0.234 |
|  | Ylmaz | 2005 | 0.605 | 0.381 |
|  | Yue | 2008 | 0.095 | 0.251 |
|  | Yue | 2005 | 0.218 | 0.354 |
| LEPR | Popko | 2007 | <0.05 | 0.483 |
|  | Hanaoka | 2008 | 0.07 | <0.05 |
|  | Huang | 2003 | 0.743 | 0.498 |
