# Supplementary material for: The Association of 5-HT2A, 5-HTT, and LEPR Polymorphisms with Obstructive Sleep Apnea Syndrome: A Systematic Review and Meta-Analysis
Source: PLoS One. 2014 Apr 22;9(4):e95856. doi: 10.1371/journal.pone.0095856 (PMC3995918; doi:10.1371/journal.pone.0095856)
Supplement: Table S2 — Methodological quality of included studies according to the NEWCASTLE-OTTAWA Quality Assessment Scale. (DOCX) [file pone.0095856.s002.docx]

Supplementary. Table2: Methodological quality of included studies according to the NEWCASTLE-OTTAWA Quality Assessment Scale

| **Study** | **Selection** | | | | **Comparability** | **Exposure** | | | **Total** |
| --- | --- | --- | --- | --- | --- | --- | --- | --- | --- |
|  | **Is the case definition adequate** | **Representativeness**  **of the cases** | **Selection of**  **controls** | **Definition of**  **controls** | **Comparability of cases and controls on the basis of the design or analysis** | **Ascertainment of exposure** | **Same method of ascertainment for cases and controls** | **Non-Response rate** |  |
| Yin G, et al | ★ | ★ | ★ | ★ | ★ | ★ | ★ | ★ | ★★★★★★★★ |
| Sakai K,  et al | ★ | ★ | / | ★ | ★ | ★ | ★ | ★ | ★★★★★★★ |
| Bayazit  YA, et al | ★ | ★ | / | / | ★ | ★ | ★ | ★ | ★★★★★★ |
| Piatto VB,  et al | ★ | ★ | / | ★ | ★ | ★ | ★ | ★ | ★★★★★★★ |
| Zhu JY,  et al | ★ | ★ | / | ★ | ★★ | ★ | ★ | ★ | ★★★★★★★★ |
| Chen HB,  et al | ★ | ★ | / | ★ | ★★ | ★ | ★ | ★ | ★★★★★★★★ |
| Yılmaz M  et al | ★ | ★ | / | / | ★ | ★ | ★ | / | ★★★★★ |
| Yue WF,  et al | ★ | ★ | / | ★ | ★★ | ★ | ★ | ★ | ★★★★★★★★ |
| Yue WF,  et al | ★ | ★ | / | ★ | ★ | ★ | ★ | ★ | ★★★★★★★ |
| Popko K,  et al | ★ | ★ | / | / | ★★ | ★ | ★ | ★ | ★★★★★★★ |
| Hanaoka M, et al | ★ | ★ | / | ★ | ★ | ★ | ★ | ★ | ★★★★★★★ |
| Huang R,  et al | ★ | ★ | / | ★ | ★★ | ★ | ★ | ★ | ★★★★★★★★ |
